# Focal Signet Ring-Like Prostate Adenocarcinoma Detected on Microultrasound in the Setting of Negative Multiparametric Magnetic Resonance Imaging: A Case Report

**DOI:** 10.7759/cureus.107545

**Published:** 2026-04-22

**Authors:** Kevin D Healey, James Blatchford, Shimron Brown, Emma Fenner, Phillip Latham, Nikhil Gopal

**Affiliations:** 1 Department of Urology, The University of Tennessee Health Science Center, Memphis, USA; 2 College of Medicine, The University of Tennessee Health Science Center, Memphis, USA

**Keywords:** androgen deprivation therapy, prostate cancer radiation therapy, prostate surgery, prostate tumors, signet ring cell adenocarcinoma

## Abstract

The ExactVU microultrasound (microUS) (Exact Imaging, Markham, ON, Canada) is a novel imaging modality that incorporates a standardized lesion classification system known as Prostate Risk Identification using Micro-Ultrasound (PRIMUS) to detect prostate cancer lesions. Here, we report a case of a 65-year-old patient diagnosed with Gleason 5+4 acinar adenocarcinoma with focal signet ring-like (mucinous) features identified on microUS that was not visualized on multiparametric magnetic resonance imaging (mpMRI). This case highlights microUS as a promising tool to improve cancer detection when used in conjunction with mpMRI, as well as the need for further research regarding unconventional prostate cancer histologic variants.

## Introduction

Prostate cancer is the most common malignancy aside from skin cancer involving men in the United States and the second most common malignancy worldwide [1]. Early detection of clinically significant prostate cancer is vital to long-term patient outcomes. Screening for prostate cancer routinely involves a prostate-specific antigen (PSA) blood test. When PSA becomes elevated, multiparametric magnetic resonance imaging (mpMRI) is often the imaging modality of choice for detecting prostate cancer lesions. Routinely, mpMRI is performed, which is then followed by a fusion biopsy. The ExactVU microultrasound (microUS) (Exact Imaging, Markham, ON, Canada) is a novel imaging modality that provides live high-resolution imaging of the prostate (29 MHz as opposed to 6-10 MHz for standard transrectal ultrasound imaging). It incorporates a standardized five-tiered lesion classification system known as Prostate Risk Identification using Micro-Ultrasound (PRIMUS), analogous to the Prostate Imaging Reporting and Data System (PI-RADS) classification for MRI [2]. MicroUS has been shown to be non-inferior compared to MRI-targeted prostate biopsy in detecting prostate cancer [2]. Rare histologic variants of prostate cancer, including tumors with signet ring-like features, represent a small but clinically important subset associated with aggressive behavior and diagnostic challenges, further complicating detection with standard imaging modalities.

## Case presentation

A 65-year-old African American male with a past medical history of erectile dysfunction (ED) and osteoarthritis was referred to our urology clinic for further evaluation of elevated PSA. At the time of his first outpatient visit, his PSA was 7.1 ng/mL, increased from 4.1 two years prior. Repeat PSA remained elevated at 7.5 ng/mL (Table 1).

**Table 1:** Prostate-Specific Antigen (PSA) Lab Value Prior to Surgery PSA values are reported in ng/mL. The reference range for normal PSA is 0.0-4.0 ng/mL. Values above this range may be associated with prostate cancer, benign prostatic hyperplasia, prostatitis, or recent urologic manipulation. A PSA value <0.1 ng/mL is considered undetectable and is typically seen following definitive treatment.

Test	Value	Reference Range
PSA	7.1	0-4 ng/mL
PSA (repeat)	7.5	0-4 ng/mL

**Figure 1:**
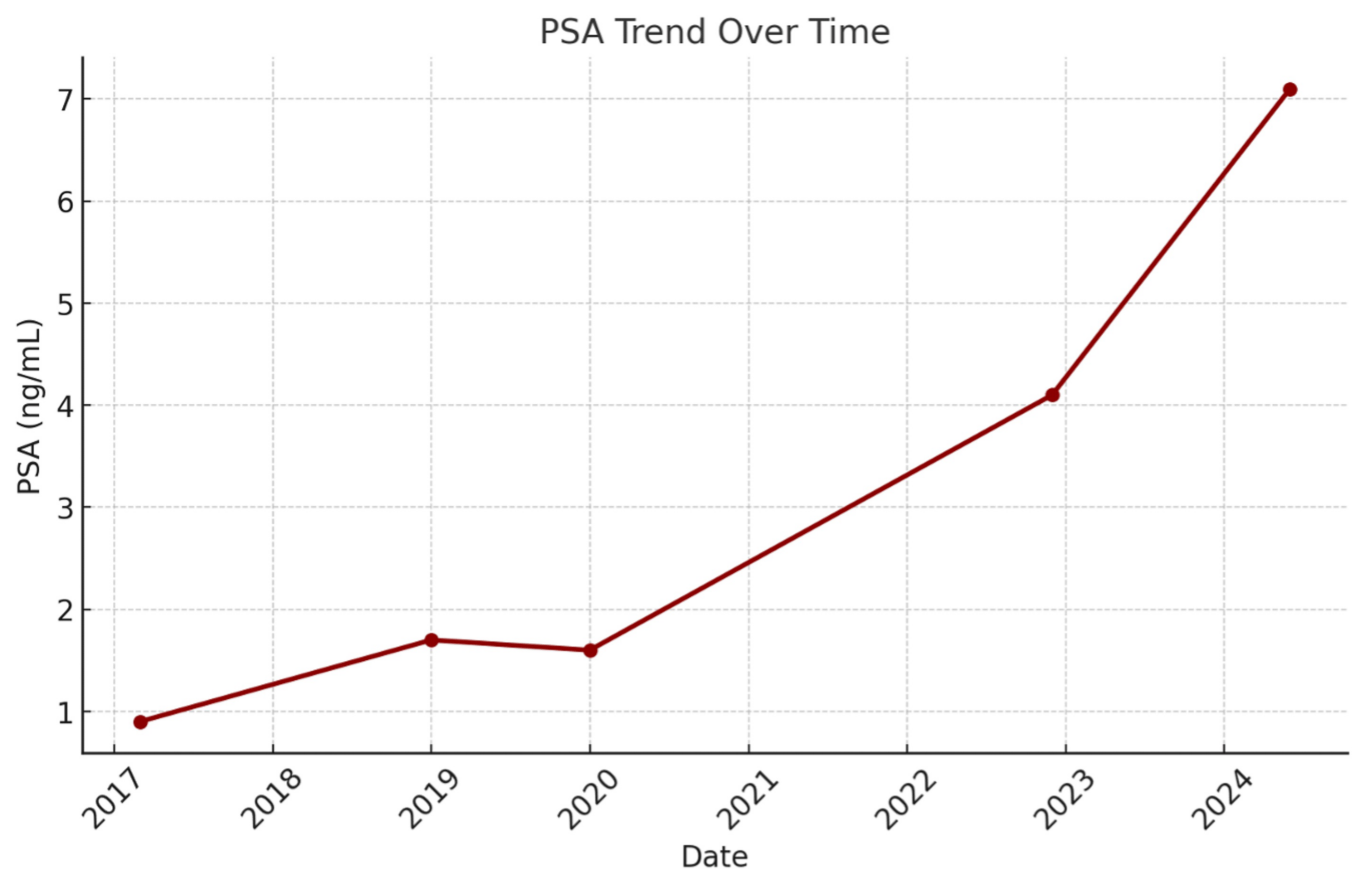
Prostate-Specific Antigen (PSA) Lab Value Trend Over Time Trend of PSA levels over time, measured in ng/mL. Rising values may indicate disease progression or recurrence, while stable or decreasing values suggest treatment response or disease control.

The patient denied any recent trauma, urethral instrumentation, hematuria, or voiding complaints, and there were no obvious signs of urinary tract infection. Urinalysis was negative for blood, nitrites, and leukocyte esterase. The patient’s PSA trend is demonstrated in Figure 1.

**Figure 2:**
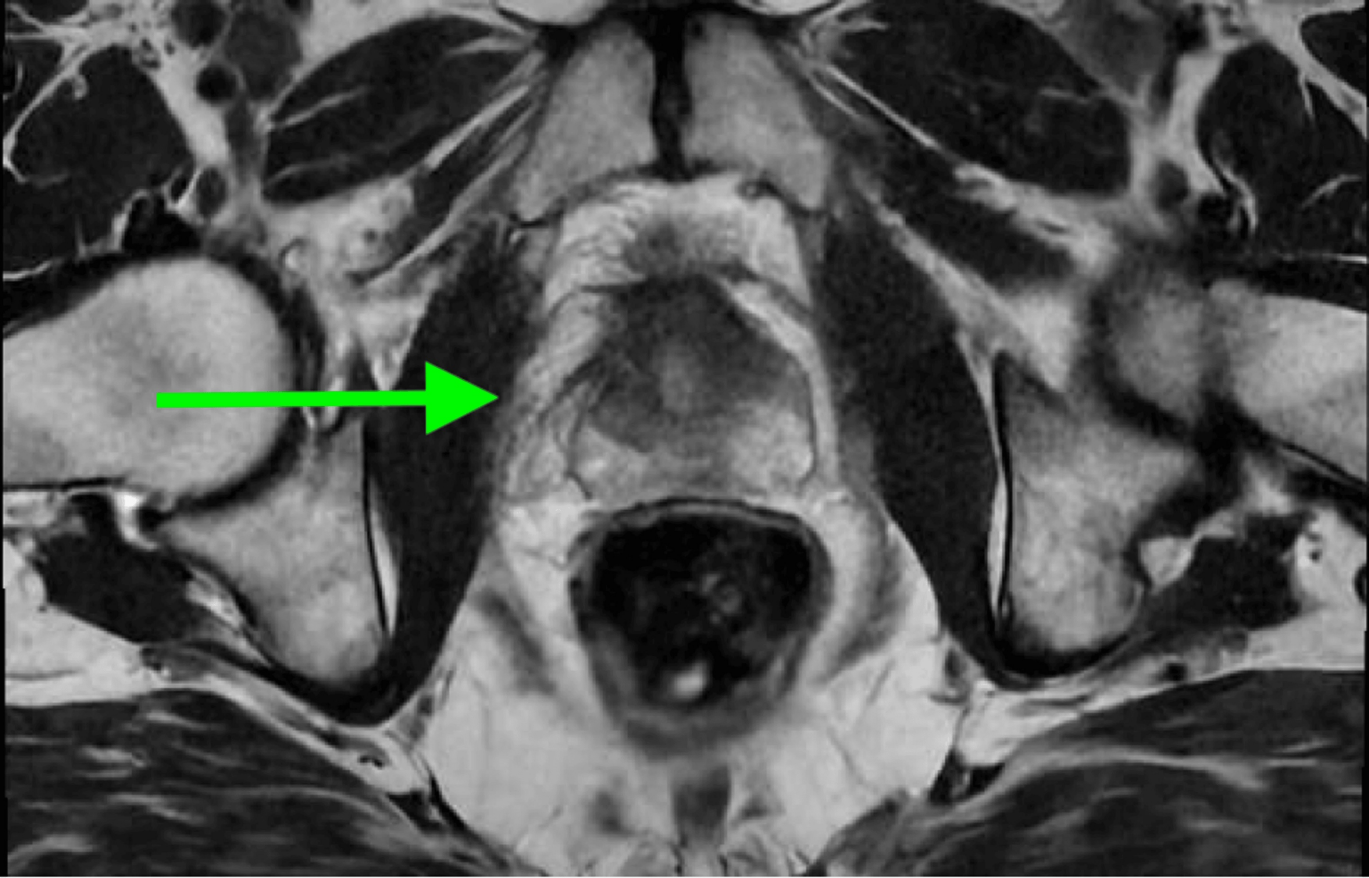
Multiparametric Prostate Magnetic Resonance Imaging (MRI), Axial T2-Weighted Image, Demonstrating No Prostate Imaging Reporting and Data System (PI-RADS) Score ≥3 Lesion No focal lesion meeting PI-RADS ≥3 criteria, suggesting no imaging evidence of clinically significant prostate cancer. The arrow points to the prostate gland.

A prostate MRI was obtained, which demonstrated a 20-gram gland and no suspicious lesions (Figure 2).

**Figure 3:**
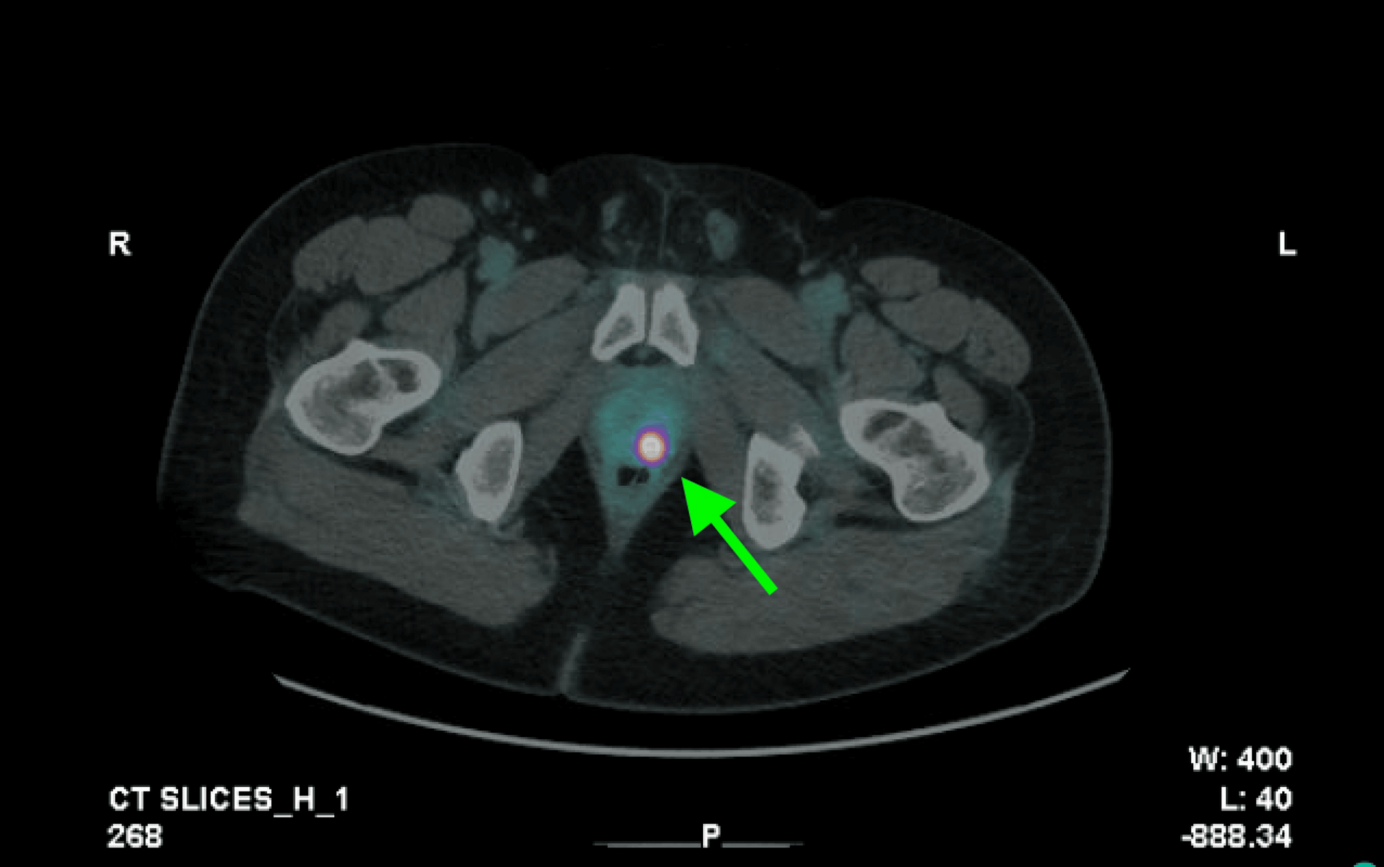
PSMA PET/CT Demonstrating Focal Uptake in the Left Posterior Prostate Axial fused gallium-68 (68Ga) prostate-specific membrane antigen (PSMA) positron emission tomography/computed tomography (PET/CT) image demonstrating focal radiotracer uptake in the left posterior prostate (arrow), corresponding to a suspicious lesion concerning for malignancy.

PSA density was calculated at 0.375 ng/mL/g, further supporting suspicion for clinically significant disease. The patient then underwent ExactVu microUS-guided systematic biopsy with 7 cores obtained from the left hemigland and 5 cores from the right hemigland. During the biopsy, a PRIMUS 4 lesion in the left peripheral zone, concerning for clinically significant malignancy, and an additional 3 cores were obtained, for a total of 15 cores. Pathology was significant for Gleason 5+4 prostate adenocarcinoma in 6/7 cores obtained from the left systematic biopsy, involving 30% of tissue with perineural invasion, 3+4 in 2/5 cores from the right systematic biopsy, involving <10% tissue without perineural invasion, and 5+4 in 2/3 cores from the PRIMUS 4 lesion, involving 50% of tissue with perineural invasion. Biopsy results are available in Table 2. Focal signet ring features were found in the PRIMUS lesion and the left prostate. A prostate-specific membrane antigen (PSMA) scan was then obtained, which revealed a focal area of increased avidity within the left posterior prostate consistent with malignancy; no extraprostatic disease was identified (Figure 3).

**Table 2:** Most Recent Prostate-Specific Antigen (PSA) Value At nine months postoperatively, PSA remained <0.1 ng/mL, reflecting an undetectable level and an appropriate biochemical response to surgical treatment.

Test	Value	Reference Range
PSA	<0.1	0-4 ng/mL

The patient elected to undergo robotic-assisted laparoscopic prostatectomy. Surgical pathology report was significant for T3aN0 disease, prostatic adenocarcinoma, Gleason 5+4 with extraprostatic extension at the left posterior aspect with an adjacent <1mm margin. Histologic subtype of the cancer was predominantly acinar adenocarcinoma with a component of signet ring-like (mucinous) features. The exact proportion of signet ring-like cells was not quantified in the pathology report. The patient was discussed in a multidisciplinary tumor board and is currently being treated with adjuvant radiation therapy to the prostate bed and pelvic lymph nodes, and two to three years of androgen therapy. Following surgery, PSA has been monitored every three months and has remained undetectable, with the most recent value at nine months post-prostatectomy also undetectable (Table 2).

## Discussion

Early detection of clinically significant prostate cancer is vital to long-term patient outcomes [1]. Once PSA has become elevated, mpMRI is the current imaging modality of choice for detecting prostate cancer lesions, with it detecting approximately 85% of Gleason Grade Group 2 or higher tumors [3]. However, mpMRI is not without limitations. An increasing body of literature has shown that mpMRI can miss clinically significant prostate cancer. False negative rates have been reported ranging from 2-18%, with most studies reporting rates closer to the latter [4,5].

mpMRI frequently misses small, low-grade, and multifocal prostate lesions, with false-negative rates reaching up to 90% for lesions under 5 mm [6]. Detection sensitivity is further compromised by specific histopathological factors such as low tumor density, heterogeneity, and infiltrative growth patterns, which often lead to missed findings in nearly half of patients with multifocal disease [6-8].

ExactVu microUS is a novel imaging modality that provides high-resolution imaging of the prostate with the PRIMUS standardized lesion and risk classification system, and is potentially more accessible and cost-effective [9]. Several studies have been conducted comparing microUS and mpMRI in the detection of prostate cancer. Single-center studies by Lughezzani et al. and Ghai et al. showed comparable sensitivities between microUS and mpMRI [10,11], which was corroborated by Hofbauer et al. in a multicenter study that showed noninferiority of microUS in detecting prostate cancer [2]. The recent OPTIMUM randomized control trial has further solidified this evidence by, once again, showing that microUS was noninferior to mpMRI in the detection of clinically significant prostate cancer. The OPTIMUM trial also found that there are lesions that are visible on only one of the imaging modalities [12]. This detection disparity has been studied previously, with research primarily focusing on how microUS can detect cancers missed by mpMRI. Avolio et al. found that the addition of microUS to mpMRI improved the diagnosis of clinically significant prostate cancer by nearly 25% and reduced the diagnosis of non-clinically significant lesions by 83% [13]. Albers et al. showed that 25% of patients with a negative mpMRI that underwent microUS-guided biopsy had clinically significant prostate cancer [14]. The case presented here further adds to the growing literature that the addition of microUS to the diagnostic arsenal can improve the detection rates of clinically significant prostate cancer and detect lesions invisible to mpMRI.

Prostate cancers harboring rare histological subtypes make up 5% of prostate cancers, and they are often underdiagnosed with current biopsy techniques, possibly since they almost always present in a mixed histological form [15]. These rare histological subtypes have a more aggressive clinical course and are more likely to recur after treatment. One such rare subtype is acinar adenocarcinoma with focal signet ring-like features as seen in this case. It is differentiated from primary signet ring-like cell carcinoma (SRC) by the percentage of signet ring-like cells in the biopsy. The 2022 World Health Organization classification states that acinar adenocarcinoma with focal signet ring-like features becomes SRC when 20% or more of the biopsy is signet ring-like cells [16]. However, there is inconsistency throughout the literature on this number, with different studies using cut-offs ranging from 5% to 50% [17,18]. Because of this lack of consensus, there is uncertainty as to the true characteristics of adenocarcinoma with signet ring-like cell features and SRC. SRC has a dismal prognosis with five-year survival rates ranging from 0% to 11%, often presenting with advanced local invasion or metastasis, and it has been reported to have unpredictable response rates to hormone treatment [17,18]. Our report is limited by the absence of microUS imaging for direct visualization of the PRIMUS lesion, as well as the lack of detailed immunohistochemical characterization of the signet ring-like component.

After treatment, PSA levels have been used for surveillance of SRC recurrence following the protocols set for typical acinar adenocarcinoma, but it has been reported that some types of SRC are PSA negative [17]. To the best of our knowledge, no protocols exist for surveillance of these cancers.

## Conclusions

This case highlights the potential role of microUS in detecting clinically significant prostate cancer not visualized on mpMRI, particularly in the setting of rare histologic variants. However, conclusions are limited by the single-case design, short follow-up duration, absence of microUS imaging, and lack of detailed immunohistochemical characterization. Further studies are needed to better define the role of microUS and optimal surveillance strategies in patients with uncommon prostate cancer subtypes.
